# Consequences of the COVID-19 Pandemic: Reduced Hemoglobin A1c Diabetes Monitoring

**DOI:** 10.1089/pop.2020.0134

**Published:** 2021-02-02

**Authors:** Maren S. Fragala, Harvey W. Kaufman, James B. Meigs, Justin K. Niles, Michael J. McPhaul

**Affiliations:** ^1^ Quest Diagnostics, Secaucus, New Jersey, USA.; ^2^ Division of General Internal Medicine, Massachusetts General Hospital, Boston, Massachusetts, USA.; ^3^ Department of Medicine, Harvard Medical School, Boston, Massachusetts, USA.; ^4^ Quest Diagnostics Nichols Institute, San Juan Capistrano, California, USA.

**Keywords:** hemoglobin A1c testing, diabetes, COVID-19

Guidelines-supported management of diabetes includes regular monitoring of glycated hemoglobin (HbA1c) with recommended testing frequencies of 2 to 4 times per year. Interruptions in HbA1c testing because of daily social or unusual societal circumstances, such as the stay-at-home orders associated with the COVID-19 pandemic, may impair diabetes management.

We examined weekly HbA1c test volumes at a large national laboratory during the COVID-19 pandemic stay-at-home orders (in March and April 2020) to test the hypothesis that the pandemic has led to decreased HbA1c monitoring. Weekly test volumes beginning March 1, 2020 were compared to mean weekly test volumes in the previous 60 weeks (January 6, 2019–February 29, 2020) in 1.9 million patients with diabetes (defined as having an ICD-10 code for type 1 or 2 diabetes in 2017 and with at least 2 HbA1c tests in 2018). This Quest Diagnostics Health Trends™ study was deemed exempt by the Western Institutional Review Board.

In the first 8 weeks of March and April 2020, HbA1c testing volume was reduced by as much as 66% compared to baseline weekly test volumes (Figure 1). From the trough at the week starting March 29, 2020, to the final week of the study, the week starting April 19, 2020, testing increased by 31%. Females had a larger initial decline than males (69% versus 62%, *P* < 0.001) but also a larger rebound after the trough (37% versus 27%, *P* < 0.001). Older patients (≥80 years) had the largest initial decline of any age group (75%, *P* < 0.001 compared to all age groups 30 years and older, not significant vs. the 74% decline in patients younger than 30) but also the largest end-of-observation rebound (56%, *P* < 0.001 vs. all individual age groups) where the rebound was only 5% for patients ages 30–39 years old, after an initial decline of 60%.

**FIG. 1.**
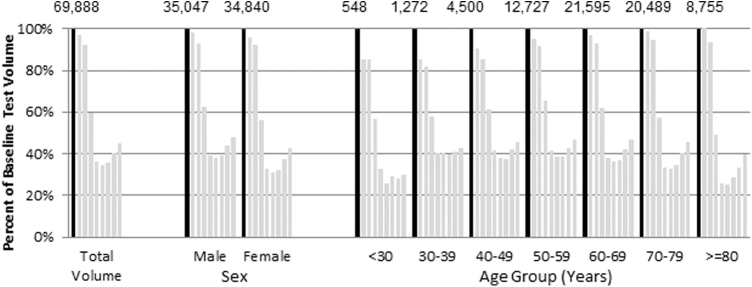
Weekly hemoglobin A1c testing among patients presumed to have diabetes, by sex and age groups. *Black bar* represents the average of baseline weekly testing volume for 60 weeks prior to March 1, 2020, for the various sex and age groupings. The numbers at the top of the chart are the baseline weekly testing volumes. The x-axis is volume in successive weeks (week 1–8) relative to baseline; March 1–7 is week 1. The y-axis depicts the weekly volumes for each category relative to the baseline weekly test volumes in percentage terms.

Reductions in HbA1c test volumes may translate to delayed testing or 1 to 2 missed tests per individual per year, depending on the duration of stay-at-home orders and avoidance of physician visits and testing events. Based on prior research, missed HbA1c monitoring appointments may be associated with HbA1c increases of 0.50%–0.83%.^1,2^ In addition, less frequent monitoring and therapy intensification also may be associated with higher blood pressure and hyperlipidemia.^2^ If unmanaged, resulting hyperglycemia may lead to a higher risk of complications–including cardiovascular disease, microvascular complications, and myocardial infarction^3^– and higher medical claims costs.^4,5^

Reductions in HbA1c testing frequency related to the COVID-19 pandemic likely reflect broader disruptions of clinical care that may be temporary or longer lasting, based on the duration of stay-at-home orders and social distancing efforts, how many patients miss testing and opportunities for intensification of therapy, and the underlying disease severity of the patients impacted. Moreover, the aforementioned potential implications were derived from populations who had different reasons for missed testing than during the pandemic. Nevertheless, the observations suggest that missed HbA1c testing during the COVID-19 pandemic could lead to significant adverse impacts in terms of glucose control, clinical outcomes, and diabetes-related medical costs.

The potential deleterious effects of interrupted diabetes monitoring highlight the importance of novel strategies to sustain good glycemic control in diabetes management. Managing blood glucose and associated risk factors (ie, blood pressure, lipids) offers savings opportunities in commercially insured populations.^5^ Control of HbA1c, blood pressure, and lipid levels has been shown to reduce the probability of diabetes-related complications by 43% to 67% in a commercially insured population,^5^ equating to cost savings of $806 to $1260 per person per year.^5^ Thus, alternative solutions to increase accessibility to needed testing may help to maintain undisrupted glucose monitoring and control in individuals with diabetes. Innovative solutions may include self-collection models and virtual care for efficient delivery of and access to health care, which have demonstrated accuracy, feasibility, and patient acceptance.

## References

[1] Karter AJ, Parker MM, Moffet HH, et al. Missed appointments and poor glycemic control: an opportunity to identify high-risk diabetic patients. Med Care 2004;42:110–115 14734947 10.1097/01.mlr.0000109023.64650.73

[2] Samuels TA, Bolen S, Yeh HC, et al. Missed opportunities in diabetes management: a longitudinal assessment of factors associated with sub-optimal quality. J Gen Intern Med 2008;23:1770–1777 18787908 10.1007/s11606-008-0757-z PMC2585658

[3] Stratton IM, Adler AI, Neil HA, et al. Association of glycaemia with macrovascular and microvascular complications of type 2 diabetes (UKPDS 35): prospective observational study. BMJ 2000;321:405–412 10938048 10.1136/bmj.321.7258.405 PMC27454

[4] Gilmer TP, O'Connor PJ, Rush WA, et al. Predictors of health care costs in adults with diabetes. Diabetes Care 2005;28:59–64 15616234 10.2337/diacare.28.1.59

[5] Fitch K, Pyenson BS, Iwasaki K Medical claim cost impact of improved diabetes control for Medicare and commercially insured patients with type 2 diabetes. J Manag Care Pharm 2013;19:609–620, 620a–620d. 24074007 10.18553/jmcp.2013.19.8.609 PMC10437463

